# Pilot evaluation of a walking school bus program in a low-income, urban community

**DOI:** 10.1186/1471-2458-9-122

**Published:** 2009-05-04

**Authors:** Jason A Mendoza, David D Levinger, Brian D Johnston

**Affiliations:** 1 USDA/ARS Children's Nutrition Research Center and Academic General Pediatrics, Department of Pediatrics, 1100 Bates St, Baylor College of Medicine, Houston, TX 77030, USA; 2 Dan L Duncan Cancer Center, Baylor College of Medicine, Houston TX, USA; 3 T-Usability, 2808 NW 92nd St, Seattle, WA 98117, USA; 4 Department of Pediatrics, University of Washington and Harborview Medical Center, 325 Ninth Ave, Seattle, WA 98104-2499, USA

## Abstract

**Background:**

To evaluate the impact of a walking school bus (WSB) program on student transport in a low-income, urban neighborhood.

**Methods:**

The design was a controlled, quasi-experimental trial with consecutive cross-sectional assessments. The setting was three urban, socioeconomically disadvantaged, public elementary schools (1 intervention vs. 2 controls) in Seattle, Washington, USA. Participants were ethnically diverse students in kindergarten-5^th ^grade (aged 5–11 years). The intervention was a WSB program consisting of a part-time WSB coordinator and parent volunteers. Students' method of transportation to school was assessed by a classroom survey at baseline and one-year follow-up. The Pearson Chi-squared test compared students transported to school at the intervention versus control schools at each time point. Due to multiple testing, we calculated adjusted p-values using the Ryan-Holm stepdown Bonferroni procedure. McNemar's test was used to examine the change from baseline to 12-month follow-up for walking versus all other forms of school transport at the intervention or control schools.

**Results:**

At baseline, the proportions of students (n = 653) walking to the intervention (20% +/- 2%) or control schools (15% +/- 2%) did not differ (*p *= 0.39). At 12-month follow up, higher proportions of students (n = 643, *p *= 0.001)) walked to the intervention (25% +/- 2%) versus the control schools (7% +/- 1%). No significant changes were noted in the proportion of students riding in a car or taking the school bus at baseline or 12-month follow up (all *p *> 0.05). Comparing baseline to 12-month follow up, the numbers of students who walked to the intervention school increased while the numbers of students who used the other forms of transport did not change (*p *< 0.0001). In contrast, the numbers of students who walked to the control schools decreased while the numbers of students who used the other forms of transport did not change (*p *< 0.0001).

**Conclusion:**

A WSB program is a promising intervention among urban, low-income elementary school students that may promote favorable changes toward active transport to school.

**Trial Registration:**

ClinicalTrials.gov NCT00402701

## Background

Childhood obesity is a major public health problem in the United States [1]. Increasing children's physical activity has been shown to decrease obesity [2] and is a major goal for Healthy People 2010 [3]. Walking to school is a promising form of physical activity that has the potential to make population-level changes to improve children's health. Walking to school is associated with higher levels of overall physical activity [4-7], is consistent with obesity prevention recommendations [8,9], and is one of the objectives for children in Healthy People 2010 [3]. Greater numbers of children walking to school in organized programs may decrease motor vehicle traffic and carbon emissions, lower risk of pedestrian injury, and lower air pollution around schools. However, several US studies have reported substantial national declines in children walking to school [10-12]. For example, in national samples of children, only 17% walked to or from school at least once a week in 2004 [11] compared to the almost 50% of elementary school children who walked or biked to school in 1969–1970 [13]. Reasons for the decline in children walking to school are likely related to increased distance from home to school, changes to the built environment, and parental concerns [14]. Parents' concern about their children's safety (traffic and crime-related) in particular was cited as the most important barrier to allowing their children to walk to school [11,14,15].

A walking school bus (WSB) is a group of children who walk to and from school chaperoned by responsible adults, usually parents. WSB programs address parents' safety concerns by providing a period of physical activity with adult supervision and teaching opportunities around pedestrian safety skills. The idea for a WSB reportedly originated in Australia as a practical transportation solution to promote physical activity and reduce congestion, pollution, and reliance on automobiles [16]. Parents took turns leading WSBs on different days of the week, which provided for a practical and convenient way to transport children to school. Children joined the WSB at various points along the set route. Students who lived far away were dropped off along the route to join the WSB. Heavy items were transported to school on a wagon pulled by one of the adult chaperones. The primary goal was to allow children to actively and safely commute to school. An additional goal was to foster the development of skills, confidence, and motivation to walk to school safely and independently.

The published, peer-reviewed literature on active commuting to school, particularly on walk to school intervention programs, is sparse as previously reviewed [17-19]. One study reported no change in the method of school transport between intervention schools assigned a travel coordinator and control schools in London [20]. An evaluation of an active transport intervention among Scottish students reported significant increases in the mean distance walked to school by intervention students versus control students [21]. In Marin County, California, a Safe Routes to School program that included a walking school bus component among several core activities, but lacked a control group, reported increases in students who walked, biked or carpooled to school and decreases in students transported by motor vehicle [22].

Despite the growing popularity of walk to school programs in the US [23], long-term controlled studies are lacking. We sought to help fill this gap by conducting a pilot assessment of the effect of a WSB program in a low-income community in Seattle, Washington. The main hypothesis was that a WSB program would increase the proportion of students walking to school and decrease the proportion of students driven to school by car in the short- and long-term.

## Methods

### Subjects

The Seattle Public Schools and Feet First, a pedestrian advocacy organization, obtained funding for a single WSB program from the Washington State Department of Transportation. This grant provided the opportunity to conduct a natural experiment. We conducted a controlled, quasi-experimental trial with one intervention and two control schools. Three public elementary schools were identified and recruited by Feet First and the Seattle Public Schools as potential sites for the WSB intervention, based on their diverse and socioeconomically disadvantaged populations. One intervention school was chosen by the grantees, based on having the greatest "school readiness," for the initial WSB program. School readiness was indicated by substantial support from the school principal and participation of key staff members and parents in the WSB program. The remaining two schools were placed on a wait-list for future WSB program funding and served as concurrent comparison sites.

All schools were urban, public, elementary schools in the Central District of Seattle, Washington and enrolled an ethnically diverse group of students from several socioeconomically disadvantaged neighborhoods (see Table 1) [24]. The schools did not have active Parent Teacher Organizations and parent involvement at the schools was generally low as per the schools' principals and key faculty members. The schools also had similar neighborhood attributes, such as adequate sidewalks, 1–2 major road arterials within one-block of the school boundaries, and relatively mildly graded hills. The communities were primarily mixed use urban neighborhoods.

**Table 1:** Demographics for the participating schools in the walking school bus evaluation.

	Intervention School (n = 347)	Control School 1 (n = 293)	Control School 2 (n = 180)	District-wide
Race/ethnicity (%)				

American Indian	4	0	3	2

Asian	21	12	2	23

African American	50	67	80	22

Latino	20	18	8	11

Caucasian	5	3	8	41

Gender (%)				

Female	44	43	52	49

Free or Reduced Lunch (%)	91	80	87	40

### Instruments

The primary outcome measures were the proportions of students who walked or were driven to school. These outcomes were determined on the same dates (to control for weather conditions) at all three schools by an in-classroom survey conducted by the three schools' homeroom teachers (all homerooms were surveyed). The survey was consistent with quantitative guidelines for WSB program measures [25] and adapted from the Marin County Safe Routes to School program [22]. Similar to the previous Marin County evaluation, the students were asked to raise their hands in class to indicate how they traveled to school that morning. However, instead of relying on volunteers [22], we partnered with the schools and had homeroom teachers conduct the assessment, which allowed for simultaneous, school-wide evaluations. They read from a standard script and asked their students, "How did you get to school today?" and read the following responses: (a) walked with an adult, (b) walked without an adult, (c) biked, (d) by school bus, (e) by metro bus, (f) by carpool, and (g) by car. The teachers instructed their students to raise their hands only once and ensured that the student classroom survey total matched the actual student daily attendance total. For the analyses, the "walked to school" category included students who walked with or without an adult. We combined carpool and car into one category because carpools were an infrequent method of transport.

The WSB program inauguration was in March 2005 and ran continuously through March 2006, except during school holidays and summer break. Outcomes were assessed by a series of 1-day, cross-sectional surveys at baseline (November 2004), one-month follow-up (April 2005), 6-month follow-up (November 2005, which allowed for time off for summer break), and 1-year follow-up (March 2006). Because we were interested in measuring the effect of the WSB program on usual student transportation, none of the data collection occurred on days with a planned walking school bus or walk-to-school promotion event. Given the constraints of funding and personnel for this pilot evaluation, we limited data collection to one day per assessment time point. We also used trained and certified professional educators/evaluators already working in the schools, i.e. teachers, who were not paid by the investigators, to conduct the simple classroom transportation assessments according to a standard script.

### Procedure

The intervention school was assigned a WSB coordinator who dedicated 10–15 hours per week throughout the entire evaluation period (except for summer break) on the project and was responsible for implementing and maintaining the program. The coordinator was hired and trained by Feet First, a Seattle pedestrian advocacy organization . In addition to establishing WSB routes and recruiting adult volunteers and students, the coordinator implemented school-wide activities and distributed materials on walking to school and pedestrian safety. For example, she maintained a bulletin board with walk to school and pedestrian safety materials, provided walk to school materials and WSB information in the school newsletter, arranged for classroom presentations on pedestrian safety by Seattle Police Officers, organized "Two-Feet Tuesdays" (a weekly walk to school day), and organized walking workshops and the annual walk to school community celebration. She also conducted an informal process evaluation by tracking WSB student attendance weekly and by face-to-face interviews with WSB parent leaders and volunteers. The WSB routes were chosen by Feet First, school personnel, and parents to maximize efficiency, safety, and participation. Parent leaders and volunteers for the WSB program were first identified by the school principal or staff. All WSB staff and volunteers passed a standard criminal background check. The intervention and control schools all received standard information on preferred walking routes from the Seattle Public Schools, access to a district-wide school traffic and safety committee, and assistance with school safety patrols.

### Data Analysis

We used Stata version 9 for Windows (StataCorp, College Station, TX). We used the Pearson Chi-squared test to compare students transported to school at the intervention versus control schools at each time point. We list estimates with their standard errors. The control schools' data were pooled. The unit of study was the school group, i.e. intervention versus the pooled control schools. Due to multiple testing, we calculated adjusted p-values using the Ryan-Holm stepdown Bonferroni procedure [26].

To examine the change from baseline to 12-month follow-up for walking versus all other forms of school transport at the intervention or control schools separately, we used McNemar's test statistic. We combined the other forms of school transport into one category because the Pearson chi-squared test indicated that only walking to school was significantly different between intervention and control schools. This study was approved by the University of Washington Human Subject Division and the Seattle Public Schools. This study's unique registration number for ClinicalTrials.gov was NCT00402701.

## Results

School characteristics were provided by the Seattle Public Schools for 2005 (Table 1) [24]. The schools were closely matched on the percentage of students who received free or reduced price meals and the percentage of ethnic minority students enrolled. For the baseline and all follow-up measurements, greater than 78% of students were counted at each school for the classroom surveys of school transport. The percentage of K-2^nd ^grade students sampled at the intervention and control schools did not differ at baseline (46% vs. 51%), 1-month (45% vs. 50%), 6-month (54% vs. 55%), and 12-month (50% vs. 56%) follow up.

From the process evaluation, we report that three WSBs were developed and maintained throughout the study period and each "bus" had its own set route to school from different locations in the surrounding neighborhoods. The routes ranged from approximately 0.3 to 1.5 miles long (as estimated by ) and took 15–40 minutes from start to finish. The WSBs had 1–3 specified pick-up points along each route. Additionally, the shortest WSB also briefly went door-to-door to pick up several students who were concentrated in a neighborhood housing project. The WSBs, on average, were staffed by a combination of four WSB parent leaders and three to five other parent volunteers. Due to limitations on volunteer availability, the WSBs operated once or twice a week. On average, 20–25 students regularly participated in a WSB at least once a week.

At baseline, the proportions of students who walked to school at the intervention (20% +/- 2% versus control (15% +/- 2%) schools were not significantly different (Table 2, *p *= 0.39). However, at 1-month (25% +/- 3% vs. 11% +/- 2%, *p *= 0.0012), 6-month (24% +/- 2% vs. 11% +/- 2%, *p *= 0.0011) and 12-month follow up (25% +/- 2% vs. 7% +/- 1%, *p *= 0.001), higher proportions of students walked to school at the intervention school versus control schools, respectively (Table 2).

**Table 2:** Percentage +/- standard errors (absolute counts of students by mode of transport/total students) of K-5^th ^grade students transported to school by car, walking, or school bus.*, §

	Baseline	1-month	6-month	12-month
By car				
Intervention	47 +/- 3 (132/281)	36 +/- 3 (106/291)	36 +/- 3 (116/323)	34 +/- 3 (102/303)
Controls	41 +/- 3 (152/372)	38 +/- 2 (169/447)	41 +/- 2 (168/406)	39 +/- 3 (134/340)

By walking				
Intervention	20 +/- 2 (56/281)	25 +/- 3^†^ (73/291)	24 +/- 2^†^ (79/323)	25 +/- 2^†^ (75/303)
Controls	15 +/- 2 (54/372)	11 +/- 2^†^ (51/447)	11 +/- 2^†^ (45/406)	7 +/- 1^†^ (24/340)

By school bus				
Intervention	31 +/- 3 (88/281)	36 +/- 3 (105/291)	37 +/- 3 (119/323)	39 +/- 3 (118/303)
Controls	40 +/- 3 (149/372)	45 +/- 2 (200/447)	44 +/- 2 (177/406)	49 +/- 3 (165/340)

* Unless otherwise indicated, differences were not significant using a Pearson Chi-squared test comparing intervention versus controls at the specified time point for each mode of transport. *P*-values were adjusted using the Ryan-Holm stepdown Bonferroni procedure.

^§^Other forms of school transport (metro bus and bicycle) were excluded from the table due to low percentages.

^†^*p *= 0.0012 at 1-month, *p *= 0.0011 at 6-months, and *p *= 0.001 at 12-months for differences between intervention versus controls.

The differences in the proportion of students transported by car to the intervention versus control schools (Table 2) did not differ at baseline, 1-month, 6-month, or 12-month follow up (all *p *> 0.05). Similarly, no significant changes were detected in transport by school bus at the intervention or control schools at each assessment time point (Table 2, all *p *> 0.05).

Comparing baseline to 12-month follow up, the numbers of students at the intervention school who walked to school increased while the numbers of students who used the other forms of transport did not change (Table 3, *p *< 0.0001). In contrast, the numbers of students at the control schools who walked to school decreased while the numbers of students who used the other forms of transport did not change (Table 3, *p *< 0.0001).

**Table 3:** Counts of student transportation (walking versus all others combined) at baseline and 12-month follow-up for intervention and control schools.

	Baseline	12-month
Intervention School*		
		
By walking	56	75
By all others combined	225	228

Control Schools*		
		
By walking	54	24
By all others combined	318	316

* *p *< 0.0001 by McNemar's test.

Bicycling and riding the metro bus were an infrequent method of transport (0–2%) at the intervention and control schools at all assessment time points.

## Discussion

With part-time administrative support, a walking school bus program was implemented in a low-income, ethnically diverse, urban, public elementary school. Intervention and control schools had statistically similar baseline proportions of students walking to school and these estimates were comparable to previously published national estimates [10-12]. The WSB program was associated with significantly higher proportions of students who walked to school at short and long-term follow-up, as compared to the control schools. Comparing baseline to 12-month follow up results, the WSB intervention was associated with an increased number (n = 19) of students who walked to school in contrast to the controls which had a decline in students (n = 30) walking to school. A previous intervention study reported increases in the number of students walking to school, although that study lacked control schools for comparison [22]. A short-term (10-week), quasi-experimental trial reported significant increases from baseline to immediate post-intervention in the mean distance walked to school (+555 meters, P < 0.001) and decreases in the mean distance traveled by car to school (-850.5 meters, P < 0.001) by intervention (Traveling Green) versus control students [21]. Our study builds upon these studies by reporting long-term results specifically for a WSB program with a study design that included control schools. Taken together, WSB programs may improve the numbers of children walking to school, and may improve their physical activity, which are both objectives of Healthy People 2010 [3]. While no long-term differences in school travel patterns were detected as a result of a previous randomized controlled trial [20], that study's school travel coordinator(s) offered only 16 hours of expert assistance over one school year to each school, which may not provide sufficient time to develop, implement, and sustain a long-term school-wide travel plan. In comparison, the school coordinator for this study's WSB program spent about the same amount of time each week at the intervention school.

These results may underestimate the change in proportions of students who walked to school since they reflected days without scheduled walking school buses. Alternatively, the results suggest that WSB programs may not need to operate WSB routes every school day to have an impact on school travel patterns. Moreover, since data collection was relatively comprehensive for each school on assessment days, the changes in school travel patterns reflect the WSB program's school-wide impact, not just its impact on students who regularly used the WSB program.

This pilot study has a number of limitations. First, the evaluation used a non-randomized design. However, the control schools were comparable to the intervention school and served predominantly disadvantaged, minority populations from the Central District of Seattle, Washington, which should minimize selection threats to internal validity. Second, method of transport to school was assessed publicly in the classroom by self-report from elementary school students similar to a previous study [22], which may limit validity. However, the baseline percentage of students who walked to school at both the intervention and control schools were consistent with previous national estimates [10-12], which suggests that the transportation measurement method was comparable to previous methods. While few active transport studies have reported validity of students' self report for method of transport to school [4-7,10,27-29], previous studies examining students' self reported school travel have demonstrated acceptable test-retest reliability (kappa coefficient 0.96) and validity (kappa coefficient = 0.80) compared to parental report in a sample of children aged 8–11 years [30] and high concordance for test-retest reliability (97%) and validity (97.5%) compared to parental report in a sample of children aged 9–11 years [31]. These studies suggest that child-assessed measures are reasonably valid and reliable. Third, method of transport to school was assessed by school teachers rather than research staff. Teachers were not specially trained nor informed of the study's goals or *a priori *hypotheses. Using teachers for the evaluation allowed us to efficiently utilize an experienced group of professionals. It seems unlikely that teachers would knowingly or unknowingly bias results, given the small nature of the WSB program, the infrequent and brief transport assessments, and the multitude of academic teaching demands that they faced. Fourth, we did not have repeated measures on individual subjects nor socio-demographic data. Instead, we conducted cross sectional surveys with relatively high student participation (>78%) at each assessment. Given the cross-sectional assessments, we cannot determine if new students enrolled at the intervention school were already more likely to walk to school (or vice versa at the control schools) as a competing explanation for the results, but this appears unlikely. Fifth, the intervention occurred at a single urban, public elementary school, which limits external validity. Sixth, the study involved only three schools with a small sample size, especially when the unit of analysis was the school group level, which limits the ability to detect differences in the study's outcomes. Finally, the measurements were taken on only one day per assessment point, due to constraints inherent with pilot studies and natural experiments. Ideally, transport would be assessed over multiple days, to better estimate habitual school transport and account for day to day variation.

This pilot evaluation was designed to efficiently provide useful preliminary information from a natural experiment within the constraints of a limited budget and rapid timeline, to inform more methodologically rigorous studies. Studies are needed to examine the impact of WSB programs on child pedestrian safety behaviors. Studies ideally should be long-term group randomized controlled trials, which longitudinally assess individual students and their socio-demographics; use objective and validated measures for transport and physical activity; assess changes in psychosocial constructs related to physical activity; and consider the role of the built environment in moderating the effects of WSB programs.

## Conclusion

We report the successful implementation of a WSB program specifically targeted to low-income, ethnically diverse elementary schoolchildren and the first to provide long-term results in the setting of a controlled trial. The program was associated with higher proportions of students walking to the intervention school compared to control schools at 1-month follow up that was sustained at 6- and 12-months follow up. Given the popularity of WSB programs and their promotion by national health and transportation authorities, additional research is necessary to assess WSB programs' impact on children's overall physical activity, weight status, academic achievement, and pedestrian safety.

## Competing interests

The authors declare that they have no competing interests.

## Authors' contributions

All three authors were involved in the conception and design of the study. JAM and BDJ were involved in the data collection. JAM led the data analysis. JAM drafted the manuscript and DDL and BDJ critically revised the manuscript. All authors read and approved the final manuscript.

## Pre-publication history

The pre-publication history for this paper can be accessed here:


